# EspT triggers formation of lamellipodia and membrane ruffles through activation of Rac-1 and Cdc42

**DOI:** 10.1111/j.1462-5822.2008.01248.x

**Published:** 2008-10-31

**Authors:** Richard R Bulgin, Ana Arbeloa, Jade C S Chung, Gad Frankel

**Affiliations:** Centre for Molecular Microbiology and Infection, Division of Cell and Molecular Biology, Imperial CollegeLondon SW7 2AZ, UK

## Abstract

Subversion of the eukaryotic cell cytoskeleton is a virulence strategy employed by many bacterial pathogens. Due to the pivotal role of Rho GTPases in actin dynamics they are common targets of bacterial effector proteins and toxins. IpgB1, IpgB2 (*Shigella*), SifA, SifB (*Salmonella*) and Map and EspM (attaching and effacing pathogens) constitute a family of type III secretion system effectors that subverts small GTPase signalling pathways. In this study we identified and characterized EspT from *Citrobacter rodentium* that triggers formation of lamellipodia on Swiss 3T3 and membrane ruffles on HeLa cells, which are reminiscent of the membrane ruffles induced by IpgB1. Ectopic expression of EspT and IpgB1, but not EspM, resulted in a mitochondrial localization. Using dominant negative constructs we found that EspT-induced actin remodelling is dependent on GTP-bound Rac-1 and Cdc42 but not ELMO or Dock180, which are hijacked by IpgB1 in order to form a Rac-1 specific guanine nucleotide exchange factor. Using pull-down assays with the Rac-1 and Cdc42 binding domains of Pak and WASP we demonstrate that EspT is capable of activating both Rac-1 and Cdc42. These results suggest that EspT modulates the host cell cytoskeleton through coactivation of Rac-1 and Cdc42 by a distinct mechanism.

## Introduction

Subversion and modulation of host cell signalling networks is essential for effective invasion and colonization of a wide variety of bacterial pathogens. In order to facilitate the hijacking of essential host processes many bacterial pathogens employ one or a combination of secretion systems (reviewed in Filloux *et al*., 2008). These complex machines are capable of delivering a wide variety of toxins, colonization factors and effectors either in proximity to epithelium or directly into the host cell (reviewed in Gerlach and Hensel, 2007). A wide variety of Gram-negative pathogens utilize type III secretion systems (T3SS) in order to translocate effector proteins directly from the bacterial cell into the cytoplasm of the mammalian cell (reviewed in Galan and Wolf-Watz, 2006). The translocated effector proteins are directed to distinct cellular compartments where, by interacting with indigenous proteins, they form novel complexes that modulate a variety of signalling networks for the benefit of the bacterial cell.

The medically important enteropathogenic and entero-haemorrhagic *Escherchia coli* (EPEC and EHEC) (reviewed in Nataro and Kaper, 1998), along with the murine pathogen *Citrobacter rodentium* (reviewed in Mundy *et al*., 2005), are extracellular bacterial pathogens, which intimately adhere to host enterocytes causing distinctive attaching and effacing (A/E) lesions characterized by the local disruption of the brush border microvili (Knutton *et al*., 1987). Colonization and persistence of EPEC, EHEC and *C. rodentium* is dependent upon a T3SS-encoded within the locus of enterocyte effacement (LEE) (McDaniel *et al*., 1995). This T3SS translocates a plethora of effector proteins that are located both within the LEE and on a variety of other pathogenicity islands and prophages (Tobe *et al*., 2006). EPEC, EHEC and *C. rodentium* translocate their own receptor, Tir (Kenny *et al*., 1997), into mammalian cells that binds the bacterial outer membrane adhesisn intimin (reviewed in Frankel *et al*., 2001), resulting in Tir clustering and formation of actin-rich pedestals beneath adherent bacteria *in vitro* (reviewed in Frankel and Phillips, 2008). Other A/E bacterial effectors that target and modulate the host cell cytoskeleton include EspG and EspG2, which disrupt the host cell microtubule network (Matsuzawa *et al*., 2004; Shaw *et al*., 2005), Map, which induces transient filopodia formation (Kenny and Jepson, 2000) and EspM, which activates the small GTPase RhoA and induces formation of stress fibres (Arbeloa *et al*., 2008).

Small GTPases act as molecular switches that cycle between an inactive GDP bound form and an active GTP bound form. The switch from inactive to active forms of the GTPase results in a conformational change. This process is regulated by a variety of accessory proteins. Guanine exchange factors (GEFs) activate GTPases by promoting the dissociation of GDP and the binding of GTP, GTPase-activating proteins (GAPs) inactivate the small GTPases by stimulating their intrinsic GTPase activity. Guanine dissociation inhibitor (GDI) proteins cap the small GTPases preventing the dissociation of GDP and membrane localization (reviewed in Jaffe and Hall, 2005). Rho GTPases modulate a variety of host cell processes in a GTP-dependent manner by activating a plethora of downstream effectors at specific host cell compartments (Stebbins and Galan, 2001). The three best characterized Rho GTPases are RhoA, Rac-1 and Cdc42, which are implicated in formation of stress fibres, lamellipodia and filopodia respectively (Jaffe and Hall, 2005).

Recently, Alto and co-workers grouped together several previously known T3SS effector proteins which share a conserved WxxxE motif and induce the same actin structures as active Rho family GTPases (Alto *et al*., 2006). These effectors include IpgB1 and IpgB2, which are required during *Shigella* invasion (Ohya *et al*., 2005), SifA and SifB which are involved in maintenance of the *Salmonella* containing vacuole (Beuzon *et al*., 2000) and Map. In addition, we recently identified EspM effectors in EPEC, EHEC and *C. rodentium*, which share significant homology with IpgB2 (Arbeloa *et al*., 2008). Using IpgB1, IpgB2 and Map, Alto *et al*. (2006) showed that the activity of these proteins in modulating actin dynamics requires the conserved tryptophan and glutamic acid residues. They also demonstrated that the formation of stress fibres induced by IpgB2 and the filopodia nucleated by Map occurred independently of the activity of RhoA and Cdc42 respectively. It was suggested that IpgB1 mimics Rac-1, IpgB2 mimics RhoA and Map mimics Cdc42 (Alto *et al*., 2006). However, a recent report by Handa *et al*. (2007) demonstrated that IpgB1 binds to the RhoG effector ELMO which in turn recruits the GEF Dock180 and subsequently activates endogenous Rac-1 to produce the membrane ruffles, while we demonstrated that EspM-induced stress fibre formation is dependent on the activation of RhoA (Arbeloa *et al*., 2008). Browsing the ongoing genome sequencing projects of A/E pathogens we recently identified a novel WxxxE effector gene, we named *espT*, in *C. rodentium*. The aim of this study was to conduct phenotypic and functional analysis of EspT.

## Results

### Identification of EspT

**Fig. 1:**
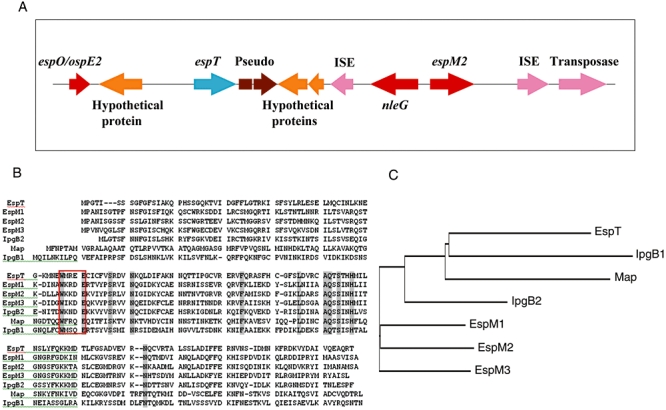
A. A graphical representation of the *espT* pathogenicity island of *C. rodentium*. B. Multiple sequence alignment with hierarchical clustering of *Shigella flexneri* IpgB1 and IpgB2 along with Map, EspM2, EspM3 and EspT from *C. rodentium* and EspM1 from EPEC B171 O111:NM. Residues that are identical are highlighted in grey. The conserved WxxxE motif is boxed. C. Phenogram created using ClustalW2 showing the phylogeny of aligned WxxxE effectors.

Recently, we identified EspM1 and EspM2 in EHEC O157:H7 (strains Sakai and EDL933), TrcA and EspM1 in EPEC O111:NM (strain B171) and EspM2 and EspM3 in *C. rodentium* as members of the WxxxE family of effectors (Arbeloa *et al*., 2008). Using the blast algorithm with IpgB2 and Map as index proteins to search for new WxxxE family members within the A/E pathogen group, we identified a new putative effector, whose encoding gene we have named *espT*, in *C. rodentium* (Accession number: FM210026)*.* EspT is encoded within the same pathogenicity island as EspM2, which also encodes an EspO/OspE homologue (Fig. 1A). OspE2 is a T3SS effector, which localizes to focal adhesions and regulate cell shape upon *Shigella sonnei* infection (Miura *et al*., 2006). EspT shares 29% identity with EspM2, 27% identity with IpgB2, and 19% identity with IpgB1. A phylogram based upon multiple sequence alignment with hierarchical clustering (Mackey *et al*., 2002) shows that EspT is divergent from the EspM/IpgB2 and does not cluster well with either IpgB1 or Map (Fig. 1C).

### EspT triggers formation of lamellipodia and membrane ruffles

**Fig. 2:**
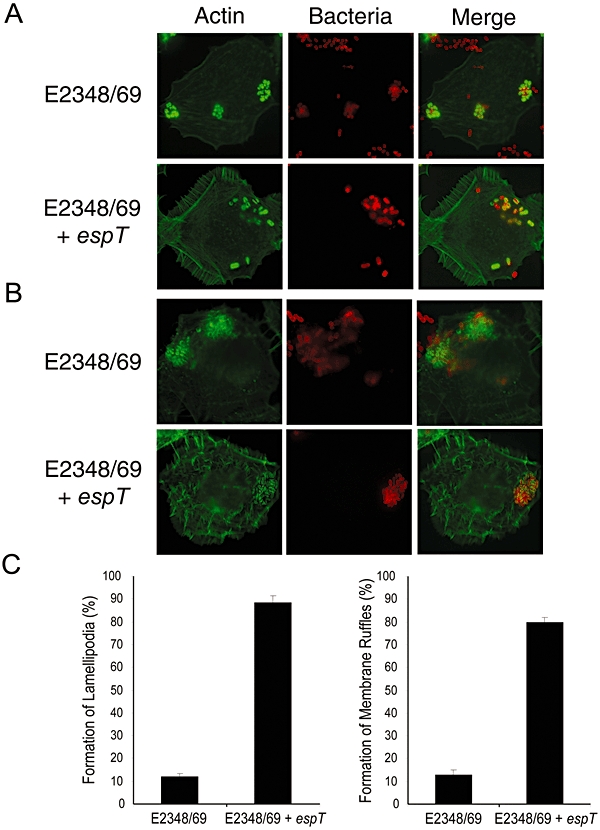
A. Fluorescence microscopy of serum-starved Swiss 3T3 cells uninfected or infected with wild-type E2348/69 or E2348/69 expressing EspT for 90 min. Actin was stained with Oregon green phalloidin and E2348/69 was detected with a rabbit O127 antibody. Distinctive lamellipodia, which have a strong leading edge, wide lamella and well defined microspikes and filopodia that extend beyond the cell boundary, were observed on cells infected with E2348/69 expressing EspT but not in the wild-type control. Both strains induced formation of actin-rich pedestals beneath adherent bacteria. B. Serum-starved HeLa cells infected with E2348/69 expressing EspT exhibited membrane ruffles whereas those infected with wild-type E2348/69 did not. Both strains formed actin pedestals. C. Quantification of Lamellipodia and membrane ruffles on Swiss 3T3 and HeLa cells, respectively, after 90 min infection with wild-type E2348/69 or E2348/69 expressing EspT. One hundred cells were counted in triplicate in three independent experiments. Results are displayed as mean ± SEM.

Previously characterized WxxxE effectors have been reported to induce a plethora of actin structures normally associated with activated Rho GTPases (Alto *et al*., 2006; Handa *et al*., 2007; Arbeloa *et al*., 2008). In order to determine if EspT has the ability to remodel actin within eukaryotic cells, *espT* was cloned into the expression vector pSA10 (Schlosser-Silverman *et al*., 2000) and expressed in EPEC E2348/69, which does not contain any WxxxE effectors other than Map (Arbeloa *et al*., 2008). T3SS-dependent translocation of EspT from E2348/69 into mammalian cells was confirmed using a β-lactamase fusion assay (data not shown). E2348/69 with or without the vector-encoding EspT was used to infect serum-starved Swiss 3T3 and HeLa cells. E2348/69 with no plasmid or carrying empty pSA10 triggered formation of actin-rich pedestals beneath adherent bacteria on both Swiss 3T3 and HeLa cells, without any other major cytoskeletal alterations (Fig. 2). In contrast, E2348/69 carrying pSA10-encoding EspT induced formation of pedestals on both cell lines and additionally triggered formation of lamellipodia on Swiss 3T3 cells and membrane ruffles on HeLa cells, which were not restricted to the site of bacterial attachment (Fig. 2A and B). The lamellipodia induced by EspT have a strong leading edge, wide lamella, well-defined microspikes and filopodia, which extend beyond the cell periphery (Fig. 2A). These lamellipodia and membrane ruffles were found in 90% and 80% of cells infected with E2348/69, respectively, after 1.5 h (Fig. 2C) and were still present at 3 h post infection. The E2348/69 type III secretion null mutant, Δ*escN*, carrying pSA10::*espT* did not produce actin-rich pedestals, lamellipodia or membrane ruffles (data not shown). These results demonstrate that EspT is a T3SS effector that upon tranlocation triggers formation of lamellipodia and membrane ruffles and that the nature of actin rearrangement was cell type-specific.

### EspT is sufficient for lamellipodia and membrane ruffle formation

**Fig. 3:**
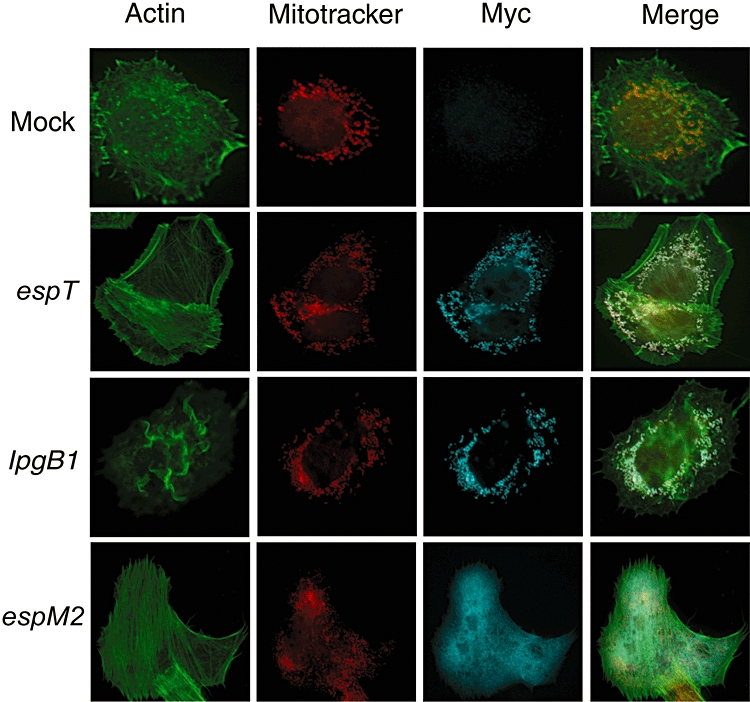
Ectopic expression of EspT induces cytoskeletal rearrangement in Swiss 3T3 cells. Serum-starved Swiss 3T3 cells were mock transfected or transfected with the mammalian expression vector pRK5 encoding myc-tagged EspT, IpgB1 or EspM2 for 16 h. Actin was stained with Alexifluor 633 phalloidin, the myc tag was detected with monoclonal antibody and mitochondria were stained with Mitotracker CMXRos. On transfection EspT induces the formation of lamellipodia identical to those observed during infection. Transfection of IpgB1 and EspM2 resulted in the induction of membrane ruffles and stress fibres respectively. EspT and IpgB1 were found in the mitochondria while EspM2 remained cytoplasmic.

Ectopic expression of *espT*, fused to a myc tag, from the mammalian expression vector pRK5 in Swiss 3T3 cells resulted in formation of lamellipodia that were indistinguishable from those visualized when EspT was delivered by E2348/69 (Fig. 3). Tranfection of EspT into HeLa cells induced formation of membrane ruffles identical to those observed during infection (data not shown). Thus, EspT is necessary and sufficient for these re-arrangements of the actin cytoskeleton. It has been previously shown that ectopic expresion of the *Shigella* effector *IpgB1* induces formation of membrane ruffles in HeLa cells (Alto *et al*., 2006; Handa *et al*., 2007). As such we transfected IpgB1, fused to a N-terminal myc tag, into HeLa and Swiss 3T3 cells as a control. In agreement with previous data (Handa *et al*., 2007), we found that IpgB1 induce membrane ruffles on HeLa cells. However, in contrast to the result we obtained for EspT, we found that IpgB1 also induces membrane ruffles in Swiss 3T3 cells (Fig. 3).

Additionally, we found that at 16 h post transfection EspT was targeted to the mitochondria, as shown by double staining with anti-myc antibodies and Mitotracker CMXRos (Fig. 3). Similarly, 16 h post transfection IpgB1 was also found in the mitochondria, while EspM2 was uniformly distributed in the cytosol (Fig. 3). Although the mitochondria appeared swollen and sometimes aggregated, the fact that they were well-stained with Mitotracker CMXRos is indicative that they were metabolically active. Moreover, we did not observed any signs of apoptosis in EspT or IpgB1 transfected cells, indicating that the mitochondrial membrane has not been compromised (data not shown). However, as EspT and IpgB1 contain no canonical mitochondrial targeting sequences, the mechanism involved in mitochondrial targeting remains unknown.

### Lamellipodia and membrane ruffles induced by EspT are dependent on activation of Rac-1 and Cdc42

**Fig. 4:**
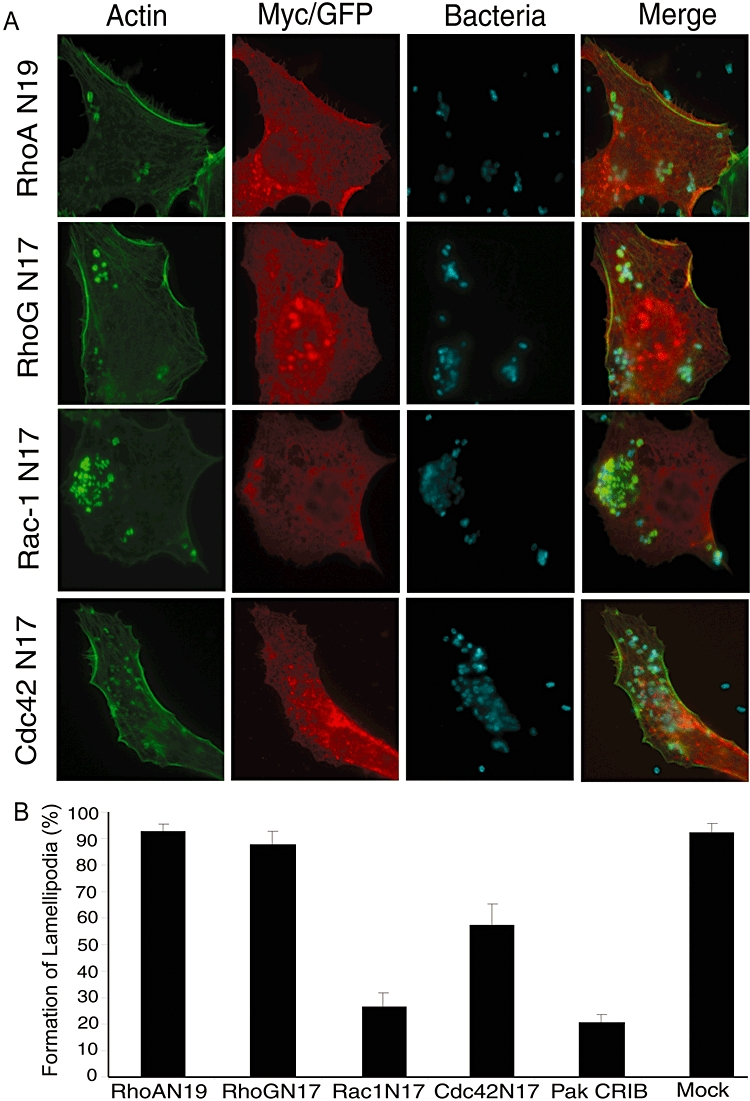
EspT-induced cytoskeletal rearrangements are dependent on Rac-1 and Cdc42. A. Swiss 3T3 cells were transfected with the dominant negative forms of RhoA, RhoG, Rac-1 and Cdc42 18 h prior to infection for 90 min with E2348/69 expressing EspT. Actin was stained with Oregon green phalloidin, the myc-tagged RhoA, Rac-1 and Cdc42 dominant negatives were stained with a myc tag monoclonal antibody, RhoG was detected via its GFP tag and E2348/69 was visualized using a rabbit O127 antibody. Actin pedestals were observed beneath all adherent bacteria. Lamellipodia were observed in cells transfected with dominant negative RhoA^N19^ and the RhoG^N17^. The majority of cells transfected with dominant negative Rac-1^N17^ did not display lamellipodia. Dominant negative Cdc42^N17^ reduced the number of cells expressing lamellipodia as well as reducing the extent of the lamellipodia compared with the mock or dominant negative RhoA^N19^ and RhoG^N17^. B. Quantification of lamellipodia in transfected Swiss 3T3 after 90 min infection with E2348/69 expressing EspT. One hundred cells were counted in triplicate in three independent experiments. Results are displayed as mean ± SEM.

WxxxE effectors have previously been demonstrated to act both independently (Alto *et al*., 2006) and dependently (Handa *et al*., 2007; Arbeloa *et al*., 2008) of the Rho GTPases. In order to determine if EspT activity was dependent on RhoA, Rac-1, Cdc42 or RhoG, we transfected the dominant negative forms of each of these GTPases (RhoA^N19^, Rac-1^N17^, Cdc42^N17^ and RhoG^N17^), which competitively and specifically inhibit the wild-type GTPase activation, into both Swiss 3T3 and HeLa cells. Additionally, we transfected the Cdc42 and Rac1 binding domain (CRIB) of Pak, which efficiently inhibits the downstream signalling of Rac1 and Cdc42 (Carreno *et al*., 2002). Transfected cell were subsequently infected with E2348/69 expressing EspT from pSA10 for 1.5 h and the presence of lamellipodia or membrane ruffles was assessed. Inactivation of either RhoA or RhoG had no effect on the induction of lamellipodia or membrane ruffles by EspT on Swiss 3T3 and HeLa cells respectively (Fig. 4A). Conversely inhibition of Rac-1 or Cdc42 significantly compromised the ability of EspT to form either lamellipodia or membrane ruffles (Fig. 4B). The residual lamellipodia observed in the presence of the Rac-1 and Cdc42 dominant negatives were shorter and less pronounced than those induced in mock transfected cells (Fig. 4A). Inhibition of both Rac1 and Cdc42 downstream signalling using the CRIB of Pak reduced the number and extent of EspT-induced lamellipodia to a level comparable with the Rac1 dominant negative (Fig. 4B). This suggests that activation and subsequent downstream signalling of Rac-1, Cdc42, or both are involved in the EspT signalling pathway.

**Fig. 5:**
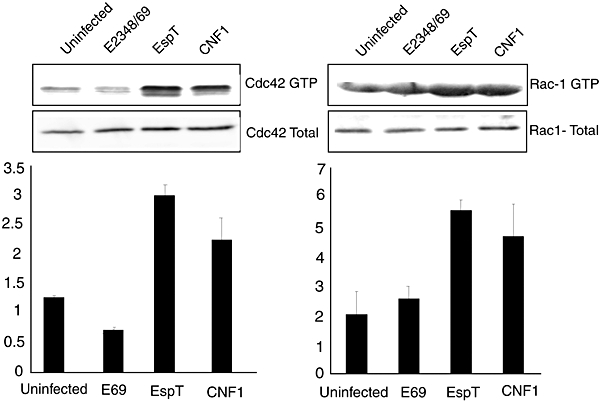
EspT activates Rac-1 and Cdc42. Swiss 3T3 cells were left untreated or infected with wild-type E2348/69 or E2348/69 expressing EspT. Cells treated with CNF1 toxin were used as a positive control. Cells were lysed and added to GST beads bound to either the CRIB of WASP or the Rac-1 binding motif of Pak to capture active Cdc42 and Rac-1 respectively. Total Cdc42 and Rac-1 along with bound Cdc42 and Rac-1 were detected by Western blotting with Cdc42 and Rac-1 monoclonal antibodies. Quantified fold activation data are presented as mean ± SEM from three independent experiments.

In order to assess whether or not EspT activates Rac-1 and Cdc42, we performed pull-down assays using the Cdc42 Rac-1 interacting binding domain (CRIB) of Pak, which binds activated Rac-1 and Cdc42 and the CRIB of WASP, which binds only activated Cdc42. Pull-downs were blotted with specific antibodies for Rac-1 and Cdc42. Cells incubated with CNF1 toxin that activates Rho GTPases (Pei *et al*., 2001) were used as a positive control, and E2348/69-containing empty pSA10 was used as a negative control. Swiss 3T3 cells infected with E2348/69 expressing EspT exhibited a marked increase in the level of activation of both Rac-1 and Cdc42 compared with uninfected cells or those infected with the control E2348/69 strain (Fig. 5B). These results suggest that EspT-triggered actin rearrangements are dependent upon activation of the Rac-1 and Cdc42 signalling cascades.

### Ectopic expression of ELMO causes a shift from lamellipodia to membrane ruffles

**Fig. 6:**
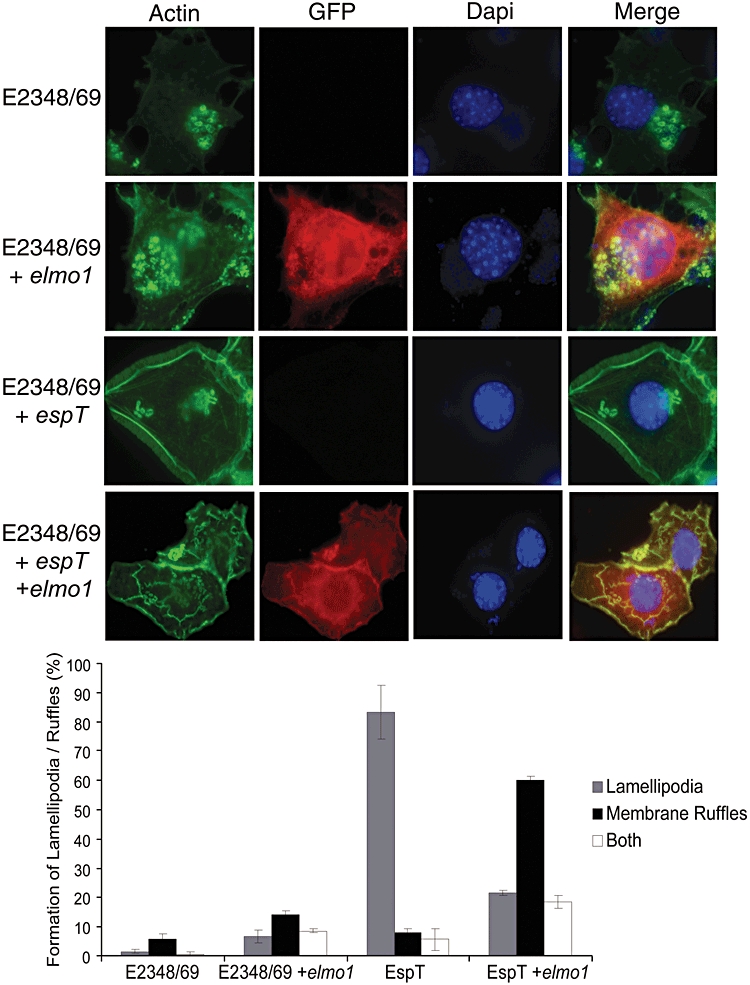
Expression of ELMO1 causes a shift from lamellipodia to membrane ruffles. A. Swiss 3T3 cells were mock transfected or transfected with GFP tagged ELMO1 18 h prior to infection with wild-type E2348/69 or E2348/69 expressing EspT. While wild-type E2348/69 displayed only pedestal formation in mock transfected or cells transfected with ELMO1, E2348/69 expressing EspT induced formation of lamellipodia on mock transfected cells but in cells transfected with ELMO there was a shift from lamellipodia to membrane ruffles. B. Quantification of Lamellipodia and membrane ruffles on mock transfected Swiss 3T3 or Swiss 3T3 cells transfected with ELMO1 after 90 min infection with wild-type E2348/69 or E2348/69 expressing EspT. One hundred cells were counted in triplicate in three independent experiments. Results are displayed as mean ± SEM.

Despite the limited homology between EspT and IpgB1, the phenotype produced by the *Shigella* effector is reminiscent of the membrane ruffles induced by EspT in HeLa cells (Ohya *et al*., 2005). Additionally, IpgB1-triggered membrane ruffles also occur in a RhoG-independent and Rac-1-dependent manner (Handa *et al*., 2007). IpgB1 has been reported to interact with ELMO1 and ELMO2 and subsequently recruit DOCK180 in order to activate Rac-1 in a manner analogous to RhoG (Handa *et al*., 2007). Furthermore, Handa and co-workers show that ELMO is localized to the membrane ruffles produced during *Shigella* invasion. In order to determine if ELMO was also localized to EspT-induced dorsal ruffles and lamellipodia, we ectopically expressed ELMO1 from the mammalian expression vector pGFP. Surprisingly, when Swiss 3T3 expressing ELMO were infected with E23348/69 carrying pSA10::*espT*, a shift from production of lamellipodia to membrane ruffles was observed with ELMO weakly localized to these ruffles (Fig. 6). These results suggest that while EspT may have a preferred binding partner in Swiss cells, it may also be able to interact directly or indirectly with ELMO. Alternatively, expression of ELMO might interfere with the pathway used by EspT to activate Rac-1 and Cdc42.

### EspT-induced cytoskeletal rearrangements are independent of ELMO or Dock180 activity

**Fig. 7:**
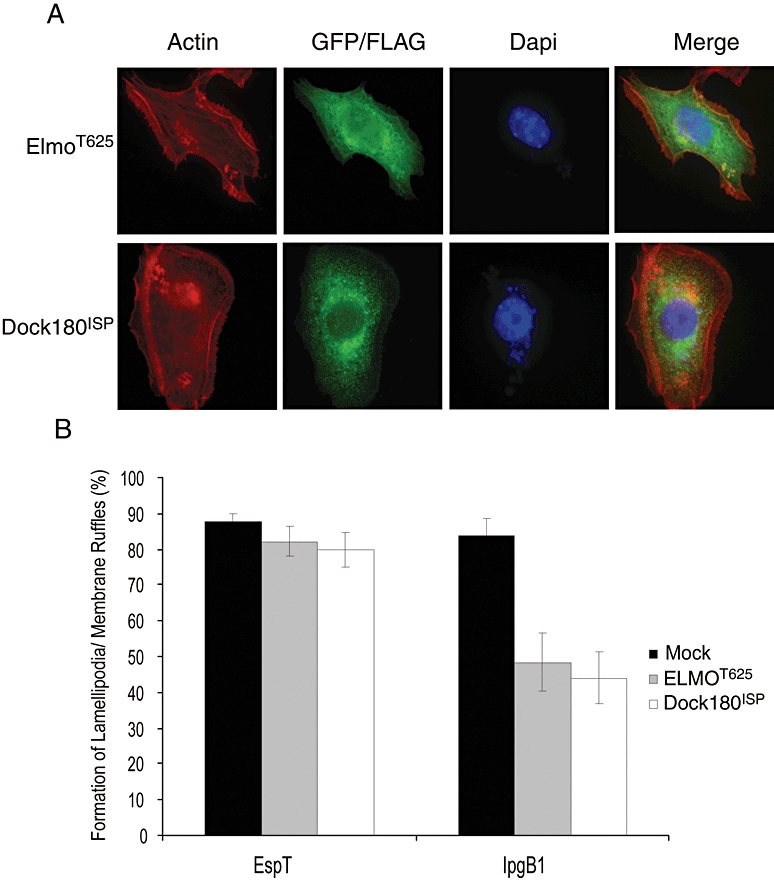
A. EspT-induced cytoskeletal rearrangements are not dependent on ELMO or Dock180. Swiss 3T3 were transfected with the dominant negative forms of ELMO and Dock180 18 h prior to infection with E2348/69 expressing EspT. Neither ELMO^T625^ nor Dock180^ISP^ had any effect on lamellipodia induction by EspT. B. Quantification of lamellipodia or membrane ruffles in Swiss 3T3 cells transfected with dominant negative ELMO^T625^ or Dock180^ISP^ and infected with E2348/69 expressing EspT or cotranfected with IpgB1 from *S. flexneri* as control. One hundred cells were counted in triplicate from three independent experiments. Results are displayed as mean ± SEM.

As ectopically expressed ELMO induces a shift from lamellipodia to membrane ruffles we utilized dominant negatives in order to investigate the role of the ELMO-Dock180 machinery in the induction of lamellipodia and membrane ruffles by EspT on Swiss 3T3 and HeLa cells respectively. Cells were transfected with ELMO^T625^ (a dominant negative of ELMO unable to interact with Dock180) or Dock180^ISP^ (a dominant negative form of Dock180 incapable of interacting with Rac-1) (Handa *et al*., 2007). Transfection of ELMO^T625^ or Dock180^ISP^ had no effect on the production of EspT-dependent lamellipodia in Swiss 3T3 cells (Fig. 7) or membrane ruffles in HeLa cells (data not shown). In contrast, double transfection of ELMO^T625^ or Dock180^ISP^ with pRK5 expressing IpgB1 resulted in a marked reduction of membrane ruffles compared with mock transfected cells (Fig. 7B). These results suggest that EspT induces lamellipodia and membrane ruffles via a mechanism that is distinct from IpgB1.

## Discussion

In this study, we identified the *C. rodentium* effector protein EspT. Previously identified effectors, Map, EspM, IpgB1 and IpgB2, which are grouped together with EspT, have all been reported to be potent modulators of the host cell cytoskeleton (Kenny and Jepson, 2000; Alto *et al*., 2006; Handa *et al*., 2007; Arbeloa *et al*., 2008). Consistent with this, using E2348/69 as a delivery system to translocate EspT along with ectopic expression we have shown that EspT also subverts host actin dynamics. Interestingly, the effect of EspT was cell-line specific. While expression in Swiss 3T3 led to formation of lamellipodia, EspT-triggered membrane ruffles on HeLa cells. When EspT was delivered by infection via E2348/69, the lamellipodia and membrane ruffles appeared as early as 15 min post infection and were stable for over 3 h.

Based on the initial examination of IpgB1, IpgB2 and Map, it was suggested that these effector proteins are molecular mimics of Rho GTPases and function in a GTP-independent manner (Alto *et al*., 2006). More recently, Handa *et al*. showed that IpgB1 activates Rac-1 via the ELMO-Dock180 machinery (Handa *et al*., 2007). In addition, we recently identified a new subfamily in the A/E pathogen group, which share a high degree of similarity to IpgB2 named EspM. We have shown that EspM2 and EspM3 activate RhoA via a mechanism which is yet to be determined (Arbeloa *et al*., 2008).

Despite EspT sharing limited sequence identity with other family members, the membrane ruffles observed on HeLa cells were reminiscent of those induced by IpgB1 (Handa *et al*., 2007) except that the EspT-induced ruffles were not restricted to the site of bacterial attachment. As IpgB1 plays a role in *Shigella* cell invasion (Ohya *et al*., 2005), we tested if overexpression of EspT affects the level of E2348/69 cell entry. Invasion assay of wild-type E2348/69 and E2348/69 expressing EspT did not reveal any significant difference in the level of intracellular bacteria (data not shown). Interestingly, expressing EspT in Swiss cells led to formation of lamellipodia, which have a strong leading edge, wide lamella and well-defined microspikes and filopodia that extend beyond the cell boundary. In contrast, IpgB1 expression induced membrane ruffles on Swiss cells.

Lamellipodia and membrane ruffles are normally regulated by interplay between Rac-1, Cdc42, RhoG and RhoA (Sander and Collard, 1999; Kurokawa *et al*., 2004; Ladwein and Rottner, 2008). In order to determine the pathway by which EspT subverts the host cell cytoskeleton, we utilized dominant negative forms of these GTPases to competitively inhibit the wild-type proteins. In the presence of dominant negative Rac-1^TN17^ a 60% reduction in formation of lamellipodia was observed. Transfection of Cdc42^TN17^ reduced lamellipodia formation by 30%. These dominant negative constructs attenuated EspT-induced membrane ruffles in HeLa cells to a similar magnitude. These results demonstrate that the actin re-arrangements induced by EspT are dependent on the activation of Rac-1 and to a lesser extent Cdc42. Furthermore, using the CRIB domains of PAK and WASP that bind activated Rac-1 and Cdc42, respectively, we demonstrate that EspT activates both Rac-1 and Cdc42 during infection of Swiss 3T3 cells. These results are consistent with the report of (Handa *et al*. (2007) who have previously demonstrated that IpgB1 activates Rac-1. We currently do not know whether EspT activates Rac-1 directly, via activation of Cdc42 or through a yet unknown GEF or adaptor.

Handa and co-workers also show that IpgB1 interacts with ELMO and subsequently recruits Dock180 to a trimeric complex which brings about the activation of Rac-1 (Handa *et al*., 2007). As EspT induces a Rac-1-dependent phenotype similar to IpgB1 in HeLa cells we investigated the role of ELMO during EspT-triggered actin modulation. We transfected a GFP-tagged ELMO into Swiss 3T3 cells and infected these cells with wild-type E2348/69 or E2348/69 expressing EspT. No ruffles were observed during E2348/69 infection. Surprisingly, when ELMO was transfected into Swiss 3T3 cells infected with E2348/69 expressing EspT, we observed a shift from the lamellipodia to membrane ruffles similar to those induced by IpgB1. This result might suggest that EspT is able to interact with ELMO. Alternatively, overexpression of ELMO could have an effect downstream of the signalling pathway employed by EspT. To determine if the lamellipodia observed in Swiss 3T3 cells or the membrane ruffles formed in HeLa cells are dependent upon the ELMO – Dock180 machinery, we used the dominant negative forms of ELMO (ELMO^T625^) and Dock180 (Dock180^ISP^), which have been demonstrated to abolish IpgB1 induced membrane ruffles (Handa *et al*., 2007). As previously observed (Handa *et al*., 2007) these dominant negatives greatly reduced the formation of membrane ruffles induced by IpgB1. However, neither the ELMO^T625^ nor Dock180^ISP^ had any significant affect on the formation of lamellipodia or membrane ruffles induced by EspT, suggesting that EspT activates Rac-1 and Cdc42 by a novel mechanism.

Map, which triggers filopodia formation via activation of Cdc42 (Berger *et al.*, 2008), contains an N-terminus mitochondrial targeting sequence (Papatheodorou *et al*., 2006), while EspM2, IpgB1 and EspT do not have any obvious mitochondrial targeting sequences. EspM2, which triggers formation of parallel stress fibres upon transfection, localizes in the cytoplasm. In contrast, we found here that EspT and IpgB1 are targeted to mitochondria following 16 h ectopic expression. It is interesting to note that mitochondrial trafficking and morphology is regulated by the atypical Rho GTPases Mitochondrial Rho (Miro) 1 and 2 (Frederick *et al*., 2004; Fransson *et al*., 2006). The mitochondrial localization of IpgB1 and EspT may suggest a role for these proteins in regulating mitochondrial dynamics as well as subversion of the host actin cytoskeleton. However, although ectopic expression of EspM leads to cytosolic localization, we cannot rule out the possibility that mitochondria targeting by EspT and IpgB1 is due to the overexpression. Further studies are needed to determine if EspT and IpgB1 have a physiological mitochondrial function.

Further work is also required to elucidate the mechanism by which EspT activates Rac-1 and Cdc42. Interestingly, although *espT* is absent from the common EPEC strains (including the prototypes E2348/69 and B171) and EHEC O157, we recently found an *espT* homologue (78% identity) in the atypical EPEC strain E110019, which caused a particularly severe outbreak in Finland in 1987 (Viljanen *et al*., 1990). However, the overall prevalence of *espT* among clinical EPEC and EHEC strain is not yet known. Finally, additional studies are required to elucidate what role EspT plays *in vivo* during infection of A/E pathogens.

## Experimental procedures

### Bacterial strains, growth conditions and cell culture

The bacterial strains used in this study are listed in Table 1. Bacteria were grown in Luria–Bertani (LB) broth at 37°C or maintained on LB plates. Culture media were supplemented with Ampicillin (100 μg ml^−1^) as appropriate.

**Table 1:** List of strains.

Strain	Description	Source/reference
E2348/69	EPEC O127:H6 wild-type	Levine *et al*. (1978)
ICC168	*Citrobacter rodentium*	Barthold *et al*. (1976)
ICC192	EPEC E2348/69Δ*escN*	Garmendia *et al*. (2004)

Bacterial cultures were primed prior to infection by growth in Dulbecco's modified Eagle's media (DMEM) 4500 mg ml^−1^ glucose supplemented with 1% mannose for 3 h (Collington *et al*., 1998) before addition of 1 mM IPTG to induce protein expression.

Swiss 3T3 cells were maintained in DMEM with 4500 mg ml^−1^ glucose and supplemented with 10% fetal calf serum (Gibco) and 4 mM Glutamax (Gibco). HeLa cells were cultured in DMEM with 1000 mg ml^−1^ glucose and supplemented with 10% fetal calf serum (Gibco) and 4 mM Glutamax (Gibco).

### Bioinformatics

A PSI-BLAST search (Altschul *et al*., 1997) was performed under default conditions using IpgB2 from *Shigella flexneri* (gi:13448971) and Map from EPEC (gi:2865296) as query sequences to search the latest version of the NCBI NR database and combined with a library of peptide sequences derived from all coding sequences ≥ 50 codons in length from the genome sequences of *C. rodentium* ICC168, EPEC B171, E110019 and EPEC E2348/69.

### Multiple alignment and phylogenetic analysis

Using the sequence alignment programme ClustalW2 (Larkin *et al*., 2007), a phenogram was constructed based upon hierarchical clustering of EspT, EspM2 and EspM3 form *C. rodentium*, EspM1 from EPEC B171, and IpgB1 and IpgB2 form *S. flexneri*.

### Plasmids and molecular techniques

Plasmids used in this study are listed in Table 2; primers are listed in Table 3. The gene encoding the effector protein *espT* was amplified by PCR from *C. rodentium* genomic DNA and cloned into pSA10 with a C-terminal HA tag using primer pairs 1 and 2 (Table 3). The gene encoding the effector protein *espT* was amplified by PCR from *C. rodentium* genomic DNA and cloned into pRK5 with an N-terminal Myc tag using primer pair 3 and 4 (Table 3). The gene encoding the effector protein *IpgB1* was amplified by PCR from *S. flexneri* genomic DNA and cloned into pRK5 with a N-terminal Myc tag using primer pairs 7 and 8 (Table 3). All constructs were verified by DNA sequencing.

**Table 3:** List of primers.

Name	Sequence
EspT-HA-F	5′-TTGAATTCATGCCGGGAACAATAAGCTCCAG-3′
EspT-HA-R	5′-TTCTGCAGTTAAGCGTAGTCTGGGACGTCGTATGGGTAGGTTCTCTGAGCCTCCTGAA-3′
EspT-pRK5-F	5′-CGCGGATCCATGCCGGGAACAATAAGCTCCAG-3′
EspT-pRK5-R	5′-CCAATGCACTGCAGTTAGGTTCTCTGAGCCTCCTGAA-3′
EspM2-pRK5-F	5′-TTTGGATCCGGAGCAATGCAAATTCTAAACAAAATAC-3′
EspM2-pRK5-R	5′-TTTGAATTCTCATCCCTGTATAGCACGCATCA-3′
IpgB1-pRK5-F	5′-TTTGGATCCGGAGCAATGCAAATTCTAAACAAAATAC-3′
IpgB1-pRK5-F	5′-CCAATGCATTGGTTCTGCAGTTAATTTGTATTGCTTTGAC-3′

**Table 2:** List of Plasmids.

Name	Description	Source/reference
pSA10	pKK177-3 with LacP	Schlosser-Silverman *et al.* (2000)
pRK5::*myc-rhoA*^*N17*^	Dominant negative RhoA	Pelletier *et al*. (2005)
pRK5::*myc-rac1*^*N19*^	Dominant negative Rac1	Pelletier *et al*. (2005)
pRK5::*myc-cdc42*^*N19*^	Dominant negative Cdc42	Pelletier *et al*. (2005)
pEGFPC1::*GFP-rhoG*^*N19*^	Dominant negative RhoG	Handa *et al*. (2007)
pEGFPC1::*elmo*^*T625*^	Dominant negative ELMO	Handa *et al*. (2007)
pCXN2::*dock180*^*ISP*^	Dominant negative Dock180	Handa *et al*. (2007)
pGex::*GST-CRIBWASP*	Cdc42 binding motif of WASP	Sander and Collard (1999)
pGex::*GST-PAK*	Rac1 and Cdc42 binding domain of PAk	Sander and Collard (1999)
pEGFPC1::*elmo*	Elmo GFP tagged	Handa *et al*. (2007)
pICC427	pSA10::*espT-HA*	This study
pICC228	pRK5::*espT-Myc*	This study
pICC429	pRK5::*IpgB1-Myc*	This study
pICC320	pRK5::*EspM2-Myc*	This study

The mammalian expression vector pRK5 containing one of Rho^N19^, Rac^N17^ or Cdc42^N17^ dominant negatives used in the transfection assays was a gift from Nathalie Lamarche-Vane. The pEGFP-C1 mammalian expression vector containing the RhoG^N17^ and ELMO^T625^ dominant negatives and Elmo1 along with the pCXN2 vector with the FLAG tagged Dock180^ISP^ were kindly supplied by Yutaka Handa and Chihiro Sasakawa (Handa *et al*., 2007). The vector pGEX expressing the Rac-1 binding domain of Pak, the CRIB of WASP and CNF1 toxin fused to GST were a gift from J. Bertoglio. The pRK5 vector encoding the CRIB of Pak fused to a myc tag was kindly provided by Dr Emmanuelle Caron (CMMI).

### Infection of Swiss 3T3 and HeLa cells with EPEC E2348/69

48 h prior to infection cells were seeded onto glass coverslips at a density of approximately 5 × 10^5^ cells per well and maintained in DMEM supplemented with 10% FCS at 37°C in 5% CO_2_. Three hours before infection, the cells were washed three times with PBS, the media replaced with fresh DMEM without FCS supplemented with 1% mannose and 500 μl of primed bacteria were added to each well and infections were carried out at 37°C in 5% CO_2_ for 1.5 h.

### Transfection

Swiss 3T3 cells or Hela cells were transfected with pRK5 encoding RhoA^N19^, Rac^N17^, Cdc42^N17^ dominant negatives fused to a Myc tag, pEGFP-C1 containing RhoG^N17^ or ELMO^T625^ dominant negatives with a GFP tag, pCNX2 containing Dock180^ISP^ dominant negative with a FLAG tag or pRK5 encoding EspT, IpgB1, EspM2 or the CRIB of Pak fused to a Myc tag by lipofectamine 2000 (Invitrogen), according to the manufacturer's recommendations. The cells were incubated at 37°C in a humidified incubator with 5% CO_2_ for 16 h, washed twice in PBS before having their media replaced with DMEM as described previously. Transfected cells were infected with the appropriate strain as described above.

### Immunofluorescence staining and microscopy

Coverslips were washed three times in PBS and fixed with 3% paraformaldehyde for 15 min before washing three more times in PBS. For immunostaining, the cells were permeabilized for 5 min in PBS 0.5% Triton X-100, washed three times in PBS and quenched for 30 min with 50 mM NH_4_Cl. The coverslips were then blocked for 1 h with PBS 0.5% BSA before incubation with primary and secondary antibodies. The primary antibody mouse αFLAG, mouse anti-HA (Cell Signalling Technology) and mouse anti-Myc (Millipore) were used at a dilution of 1:500, while rabbit anti-O127 (a gift from Dr Roberto La Ragione, VLA, UK) was used at a dilution of 1:150. Coverslips were incubated with the primary antibody for 1 h, washed three times in PBS and incubated with the secondary antibodies. Donkey anti-rabbit IgG conjugated to a Cy5 fluorophore or donkey anti-mouse IgG conjugated to a Cy3 fluorophore (Jackson laboratories) were used at a 1:200. Actin was stained using Oregon Green phalloidin or Rhodamine phalliodin (Invitrogen) at a 1:100 dilution. Mitochondria were visualized using Mitotracker CMX-Ros (Invitrogen) as recommended by the manufacturer. All dilutions were in PBS/0.5% BSA. Coverslips were mounted on slides using ProLong Gold antifade reagent (Invitrogen) and visualized by Zeiss Axioimager immunofluorescence microscope using the following excitation wavelengths: Cy3 – 550 nm, Cy5 – 650 nm and Oregon Green – 488 nm. All images were analysed using the Axiovision Rel 4.5 software.

### GST-PAK and GST-WASP Rho GTPase pull-down

An overnight culture of *E. coli* BL21 expressing pGEX encoding the Rac-1 binding domain of Pak or the CRIB of WASP was diluted 1:20 and cultured at 30°C until OD_260_ reached 0.7; the culture was induced with 1 mM IPTG and incubated for a further 4 h. The bacterial culture was aliquoted into 50 ml falcon tubes and centrifuged for 15 min at 4600 r.p.m. at 4°C and the pellets stored at −80°C. The pellets were re-suspended in lysis buffer [20% Saccharose, 10% glycerol, 50 Mm Tris pH 8, 200 mM Na_2_S_2_O_3,_ 2 mM MgCl_2_, 2 mM DTT and 1% of protease inhibitor cocktail (Sigma)] and sonicated five times for 10 s. The lysate was centrifuged for 30 min at 15 000 r.p.m. at 4°C. The cleared lysate was coupled to GST-glutathione S transferase beads (GE healthcare) for 45 min at 4°C.

Cell culture flasks of 75 cm^2^ were seeded with Swiss 3T3 cells, infected as described above and the cells lysed in 750 μl of Mg^++^ buffer [25 mM HEPES, pH 7.5, 150 mM NaCl, 1% NP-40, 10 mM MgCl_2_, 5% glycerol, 1 mM EDTA and protease inhibitors (Sigma)]. The lysate was transferred to a pre-chilled eppendorf tube and centrifuged at 14 000 r.p.m. for 5 min at 4°C. The cleared lysate was transferred to a fresh pre-chilled eppendorf tube containing 30 μl of GST-Pak-RBD or GST-WASP-CRIB beads as appropriate. The lysate was incubated with the beads for 1 h at 4°C. The suspension was washed three times in Mg^++^ buffer spinning down at 14 000 r.p.m. at 4°C between each wash. The protein was eluted from the beads by the addition of 45 μl of 2× protein loading buffer and the samples heated at 100°C for 5 min. The samples were loaded on a 15% SDS-PAGE gel. The gels were transferred to PVDF by wet transfer. The PVDF membranes were blocked overnight in TBS, 5% BSA at 4°C with gentle rocking. Each membrane was incubated in either monoclonal rabbit anti-Rac-1 (Santa Cruz) or rabbit anti-Cdc42 (Santa Cruz) as appropriate at a dilution of 1:500 in TBS 5% BSA for 24 h at 4°C with gentle rocking. The membranes were washed five times in TBS 1% Tween for 5 min and incubated for 45 min with 1:10000 dilution of goat anti-rabbit (Invitrogen) secondary antibody coupled to HRP at room temperature. The membranes were developed using ECL reagents (GE healthcare) before detection using chemiluminescence in a LAS 3000 Fugi imager.
